# Systemic inflammation markers (SII and SIRI) as predictors of cognitive performance: evidence from NHANES 2011–2014

**DOI:** 10.3389/fneur.2025.1527302

**Published:** 2025-05-09

**Authors:** Xiaoyue Wang, Qinghua Wen, Yujie Li, Huanhuan Zhu, Fengyin Zhang, Simin Li, Lin Zhan, Juan Li

**Affiliations:** ^1^Public Health School, Zunyi Medical University, Zunyi, China; ^2^School of Nursing, Guizhou University of Traditional Chinese Medicine, Guiyang, China; ^3^School of Nursing, Zunyi Medical University, Zunyi, China; ^4^NHC Key Laboratory of Pulmonary Immune-related Diseases, Guizhou Provincial People’s Hospital, Guiyang, China; ^5^Department of Nursing, Guizhou Provincial People’s Hospital, Guiyang, China

**Keywords:** cognitive performance, inflammatory markers, systemic immune-inflammation index, system-inflammation response index, NHANES

## Abstract

**Background:**

Neuroinflammation is linked to cognitive function. However, epidemiological research on two emerging inflammation markers—the systemic immune-inflammation index (SII) and the systemic inflammation response index (SIRI)—remains limited in the context of cognitive performance. This study investigates the relationship between SII, SIRI, and cognitive performance in older adults.

**Methods:**

This cross-sectional analysis included 2,194 participants from the 2011–2014 National Health and Nutrition Examination Survey (NHANES) who met eligibility criteria. Logistic regression, subgroup analysis, and restricted cubic spline modeling were used to assess the associations between cognitive performance and inflammation markers, specifically SII and SIRI.

**Results:**

After adjusting for population weights, participants with low cognitive function had an SII of 541.54 (95% CI: 360.00–796.50, *p* = 0.037) and an SIRI of 1.28 (95% CI: 0.82–2.18, *p* = 0.031). In fully adjusted models, higher levels of both SII (OR = 0.858, 95% CI: 0.856–0.859) and SIRI (OR = 0.891, 95% CI: 0.889–0.892) were significantly associated with lower odds of normal cognitive function, indicating an increased risk of cognitive impairment. Neutrophil-related markers (NC, NLR, SIRI) exhibited the strongest inverse associations. Subgroup analysis showed more consistent associations for SIRI across demographic and behavioral factors, while SII displayed fewer. RCS analysis indicated a stronger non-linear relationship for SIRI (*p* = 0.005) compared to SII (*p* = 0.018) after full adjustment.

**Conclusion:**

This study suggests a positive association between SII, SIRI, and cognitive function, with a more pronounced relationship for SIRI. These findings highlight the potential of SIRI as a novel, accessible marker for predicting cognitive impairment risk.

## 1 Introduction

Cognitive impairment (CI) refers to a decline in cognitive functions, including memory, language, attention, problem-solving, and executive function. CI is linked to diminished daily functioning, increased comorbidity risks, and long-term care dependency, placing substantial medical and societal burdens (1). With global aging, CI has become an escalating health concern among older adults, underscoring the importance of identifying and mitigating its risk factors to reduce prevalence.

The systemic immune-inflammation index (SII) and the systemic inflammation response index (SIRI) are composite inflammatory markers developed in recent years, derived from neutrophil, lymphocyte, monocyte, and platelet counts (2–4). Neutrophil count (NC) reflects acute inflammatory responses, lymphocyte count (LC) plays a central role in immune regulation, platelet count (PC) signals both coagulative and inflammatory activities, and monocyte count (MC) is involved in immune surveillance. Initially proposed for liver cancer prognosis (5), SII has since been explored in various contexts, while SIRI was introduced to predict post-chemotherapy survival in patients with cancers (6), with subsequent research linking elevated SIRI to lymphovascular invasion (7). Given the critical role of inflammation in chronic diseases, easily obtainable hematological indices from routine blood tests (8, 9) are widely used to assess systemic inflammation (10, 11). Common markers include the neutrophil-to-lymphocyte ratio (NLR), platelet-to-lymphocyte ratio (PLR), and monocyte-to-lymphocyte ratio (MLR). However, limited research has examined the relationship between emerging inflammatory markers like SII and SIRI and cognitive function. Investigating these associations may provide early diagnostic insights, elucidate inflammation-related neurobiological mechanisms, and offer practical biomarkers for cognitive performance assessment.

## 2 Methods

### 2.1 Data source

This study employed cross-sectional data from the NHANES, spanning three consecutive cycles: 2011–2012 and 2013–2014. Conducted by the National Center for Health Statistics (NCHS) and the Centers for Disease Control and Prevention (CDC), NHANES assesses the health and nutritional status of individuals across various age groups in the U.S., from children to older adults. All NHANES protocols were approved by the NCHS Ethics Review Board, and informed consent was obtained from each participant.

### 2.2 Study population

**Figure 1:**
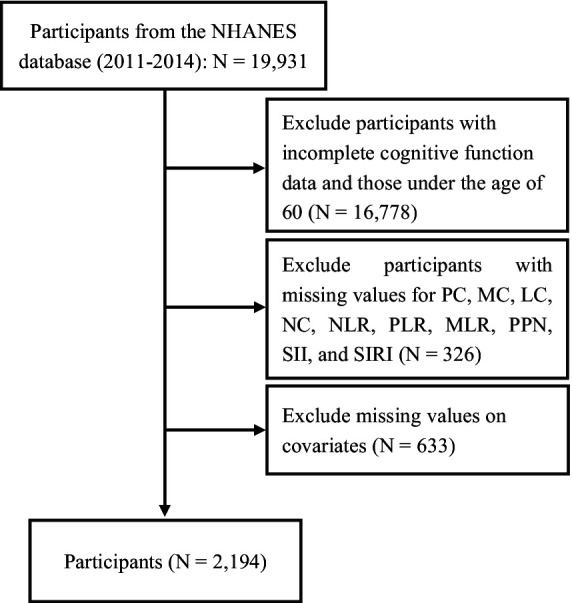
Flowchart of participant selection from NHANES 2011–2014. PC, Platelet count; MC, Monocyte count; LC, Lymphocyte count; NC, Neutrophil count; NLR, Neutrophil-to-lymphocyte ratio; PLR, Platelet-to-lymphocyte ratio; MLR, monocyte-to-lymphocyte ratio; PPN, Product of platelet count and neutrophil count; SII, Systemic immune inflammation index; SIRI, System inflammation response index.

Data from two NHANES cycles (2011–2012 and 2013–2014) were retrieved, including information from 19,931 participants. A total of 3,153 participants aged ≥ 60 years were included as they completed all cognitive function assessments. Participants with missing data for SII or SIRI measurements (*N* = 326) or covariates (*N* = 633) were excluded. Ultimately, 2,194 participants were included in the analysis (Figure 1).

### 2.3 Immune-inflammation index

SII and SIRI were calculated based on complete blood count (CBC) laboratory results from the NHANES database. These calculations utilized separate measurements of PC, NC, MC, and LC, reported in units of 1,000 cells per μL (4, 12, 13). The indices were derived as follows:

SII = (platelet count × neutrophil count)/lymphocyte count;

SIRI = (neutrophil count × monocyte count)/lymphocyte count.

To further explore the relationship between inflammation indices and cognitive performance, additional markers such as PLR, NLR, the product of platelet and neutrophil count (PPN), and MLR were also analyzed (14, 15). Given the skewed distribution of these biomarkers, Log2 transformations were applied to PC, MC, LC, NC, NLR, PLR, MLR, PPN, SII, and SIRI for regression analysis, with the transformed values used in subsequent statistical evaluations (16–18).

### 2.4 Cognitive function assessment

Cognitive performance in participants aged ≥ 60 years was evaluated using three standardized tests: (1) the Consortium to Establish a Registry for Alzheimer’s Disease Test, comprising the Immediate Recall Test (CERAD-IR) and Delayed Recall Test (CERAD-DR); (2) the Animal Fluency Test (AFT); and (3) the Digit Symbol Substitution Test (DSST). These instruments are extensively used in cohort studies on cognitive function and its risk factors (19–21). A composite Z-score, referred to as global cognitive performance (GCP), was calculated by averaging the standardized scores from the CERAD, AFT, and DSST tests (22, 23). Participants were classified into two groups—normal cognitive ability and low cognitive ability—using the median of the total Z-score as a threshold.

### 2.5 Data covariates

Continuous covariates included in this study were age, diastolic blood pressure (DBP, mmHg), systolic blood pressure (SBP, mmHg), total cholesterol (mg/dL), white blood cell count (WBC, 1000 cells/μL), and red blood cell count (RBC, 1000 cells/μL). Categorical covariates comprised sex (male, female); age categories (60–69, 70–79, ≥ 80); race/ethnicity (Mexican American, Non-Hispanic Black, Non-Hispanic White, Other Hispanic, and other races including multiracial); marital status (married/living with partner, widowed/divorced/separated, never married); education level (below high school, high school graduate, above high school); poverty-income ratio (PIR; low < 2.23, middle 2.24–4.28, high > 4.29); body mass index (BMI) categories (underweight < 18.5, normal 18.5–24.9, overweight 25–29.9, obese ≥ 30); smoking status (ever smoked at least 100 cigarettes in lifetime); alcohol consumption (defined as drinking at least 12 alcoholic drinks per year); stroke history; diabetes status based on multiple criteria, including a confirmed diagnosis, medication or insulin use, HbA1c ≥ 6.5%, fasting blood glucose ≥ 7.0 mmol/L, or a two-hour post-OGTT blood glucose ≥ 11.1 mmol/L; depression (PHQ-9 score > 10), as per previous validations (24); sleep disorder; and overall health status (categorized as excellent/very good/good or fair/poor) (25–28).

### 2.6 Statistical analysis

Statistical analyses were performed with adjustments for the complex survey design, incorporating sample weights as per CDC guidelines. The study weight was derived by halving the current year’s weight. Analyses initially included only participants with complete data on exposures and outcomes. Continuous variables were summarized as means with standard deviations, and categorical variables as percentages. Independent t-tests assessed continuous variables, while chi-square tests evaluated categorical variables. A multivariable logistic regression model was then constructed to examine the independent associations between SII, SIRI, other inflammatory markers, and low cognitive performance across three models. Subgroup analyses were conducted based on sociodemographic and lifestyle factors. The restricted cubic spline (RCS) method was employed to explore the potential non-linear relationship between SII, SIRI, and cognitive performance. All analyses were executed using R and SPSS software, with a significance level set at *p* < 0.05.

## 3 Results

### 3.1 General characteristics of the study population

A total of 2,194 participants were included in this study, categorized into two groups based on Global Cognitive Performance (GCP): low cognitive performance (*n* = 238) and normal cognitive performance (*n* = 1,956). Demographic and clinical characteristics were compared between the groups, revealing significant differences (*p* < 0.05) in age, race, marital status, education level, PIR, stroke history, diabetes, depression, general health status, SBP, RBC, WBC, NC, NLR, MLR, and SII. Of the 2,194 participants, 1,105 were male (50.36%) and 1,089 were female (49.64%). The majority of participants were aged 60–69 years (54.38%), though a notably higher proportion of those aged ≥ 80 years was observed in the low cognitive performance group (30.67%). These results indicate that cognitive decline is strongly linked to socioeconomic and health-related factors, with systemic inflammation potentially playing a significant role. Detailed results are shown in Table 1.

**Table 1:** Weighted baseline characteristics of the study participants categorized by cognitive performance status.

Participants’ characteristics	Global cognitive performance			
N	Total (2194)	Low (238)	Normal (1956)	*p*-value
Age (years)	68.00 (63.00, 74.00)	76.00 (69.00, 80.00)	67.00 (63.00, 74.00)	**<** 0**.001**
Sex, *n* (%)				0.477
Female	1,089 (52.88)	111 (56.00)	978 (52.65)	
Male	1,105 (47.12)	127 (44.00)	978 (47.35)	
Age, *n* (%)				**< 0.001**
60–69	1,193 (57.05)	79 (25.09)	1,114 (59.41)	
70–79	649 (29.04)	86 (35.00)	563 (28.59)	
> = 80	352 (13.91)	73 (39.91)	279 (11.99)	
Race, *n* (%)				**< 0.001**
Mexican American	172 (2.94)	27 (7.14)	145 (2.63)	
Non-Hispanic Black	221 (3.50)	37 (8.89)	184 (3.10)	
Non-Hispanic White	1,106 (81.39)	86 (64.00)	1,020 (82.67)	
Other Hispanic	501 (7.56)	74 (16.35)	427 (6.91)	
Other race including multiracial	194 (4.61)	14 (3.62)	180 (4.69)	
Marital status, *n* (%)				**0.016**
Married/living with partner	1,295 (66.06)	118 (52.99)	1,177 (67.03)	
Widowed/Divorced/Separated	780 (29.64)	113 (42.33)	667 (28.70)	
Never married	119 (4.30)	7 (4.68)	112 (4.27)	
Education level, *n* (%)				**< 0.001**
Below high school	518 (14.93)	132 (39.80)	386 (13.09)	
High School graduate	518 (21.39)	61 (33.82)	457 (20.47)	
Above high school	1,158 (63.69)	45 (26.38)	1,113 (66.44)	
PIR, *n* (%)				**< 0.001**
Low (< 2.23)	542 (13.79)	108 (34.66)	434 (12.25)	
Middle (2.24–4.28)	1,103 (50.17)	105 (51.24)	998 (50.09)	
High (>4.29)	549 (36.04)	25 (14.11)	524 (37.66)	
BMI, *n* (%)				**0.006**
Underweight (<18.5)	30 (1.24)	6 (2.89)	24 (1.12)	
Normal (18.5–25)	550 (24.52)	65 (34.47)	485 (23.78)	
Overweight (25–30)	788 (36.91)	85 (32.80)	703 (37.22)	
Obese (≥30)	826 (37.32)	82 (29.85)	744 (37.88)	
Smoke, *n* (%)				0.805
Yes	1,122 (50.69)	128 (49.78)	994 (50.76)	
No	1,072 (49.31)	110 (50.22)	962 (49.24)	
Alcohol, *n* (%)				**< 0.001**
Yes	1,528 (73.62)	150 (60.60)	1,378 (74.58)	
No	666 (26.38)	88 (39.40)	578 (25.42)	
Stroke, *n* (%)				**0.003**
Yes	146 (5.94)	35 (12.67)	111 (5.44)	
No	2048 (94.06)	203 (87.33)	1845 (94.56)	
Diabetes, *n* (%)				**0.006**
Yes	721 (26.65)	100 (35.26)	621 (26.02)	
No	1,473 (73.35)	138 (64.74)	1,335 (73.98)	
Depression, *n* (%)				**< 0.001**
Yes	171 (6.31)	37 (13.77)	134 (5.76)	
No	2023 (93.69)	201 (86.23)	1822 (94.24)	
Sleep disorder, *n* (%)				0.272
Yes	266 (11.97)	21 (9.40)	245 (12.17)	
No	1928 (88.03)	217 (90.60)	1711 (87.83)	
General health condition, *n* (%)				**< 0.001**
Excellent/Very good/Good	1,623 (82.14)	125 (58.56)	1,498 (83.89)	
Fair/Poor	571 (17.86)	113 (41.44)	458 (16.11)	
DBP, M (Q₁, Q₃) (mmHg)	69.33 (61.33, 76.00)	68.00 (60.00, 73.33)	69.33 (62.00, 76.00)	0.093
SBP, M (Q₁, Q₃) (mmHg)	128.67 (117.33, 140.67)	139.33 (124.67, 148.67)	128.00 (117.33, 140.00)	**< 0.001**
Total cholesterol, M (Q₁, Q₃) (mg/dL)	190.00 (161.00, 220.00)	185.00 (155.00, 211.00)	190.00 (162.00, 221.00)	**0.035**
RBC, M (Q₁, Q₃) (1,000 cells/μL)	4.51 (4.22, 4.81)	4.34 (4.01, 4.75)	4.51 (4.25, 4.81)	**< 0.001**
WBC, M (Q₁, Q₃) (1,000 cells/μL)	6.60 (5.60, 8.00)	7.00 (5.90, 8.60)	6.60 (5.60, 8.00)	0.061
PC, M (Q₁, Q₃) (1,000 cells/μL)	219.00 (184.00, 255.00)	212.00 (176.00, 256.00)	219.00 (185.00, 255.00)	0.260
MC, M (Q₁, Q₃) (1,000 cells/μL)	0.50 (0.40, 0.70)	0.50 (0.40, 0.70)	0.50 (0.40, 0.70)	0.959
LC, M (Q₁, Q₃) (1,000 cells/μL)	1.80 (1.40, 2.20)	1.70 (1.40, 2.20)	1.80 (1.40, 2.20)	0.122
NC, M (Q₁, Q₃) (1,000 cells/μL)	3.90 (3.10, 5.00)	4.40 (3.20, 5.70)	3.90 (3.10, 4.90)	**0.013**
NLR, M (Q₁, Q₃)	2.19 (1.62, 2.95)	2.58 (1.84, 3.41)	2.18 (1.61, 2.91)	**< 0.001**
PLR, M (Q₁, Q₃)	121.18 (97.08, 153.57)	121.67 (96.40, 158.46)	121.18 (97.08, 153.33)	0.594
MLR, M (Q₁, Q₃)	0.30 (0.23, 0.39)	0.31 (0.25, 0.41)	0.30 (0.23, 0.39)	0.175
PPN, M (Q₁, Q₃)	844.20 (624.00, 1180.00)	971.70 (597.60, 1384.50)	838.50 (624.00, 1168.40)	0.131
SII, M (Q₁, Q₃)	464.06 (343.33, 680.65)	541.54 (360.00, 796.50)	461.35 (342.50, 675.00)	**0.037**
SIRI, M (Q₁, Q₃)	1.16 (0.80, 1.77)	1.28 (0.82, 2.18)	1.15 (0.80, 1.73)	**0.031**

PIR, poverty income ratio; BMI, body mass index; SBP, systolic blood pressure; DBP, diastolic blood pressure; TCH, total cholesterol; PC, platelet count; NC, neutrophil count; MC, monocyte count; LC, lymphocyte count; PLR, platelet to lymphocyte ratio; NLR, neutrophil to neutrophil ratio; PPN, product of platelet count and neutrophil count; MLR, monocyte to lymphocyte count ratio; SII, systemic immune-inflammation index; SIRI, systemic inflammatory response index. Bold *p*-values indicate statistical significance at *p* < 0.05.

### 3.2 Association between SII, SIRI, and cognitive performance

Table 2 presents the associations between Log2-transformed SII, SIRI, and other inflammatory markers and cognitive performance. Several inflammatory indices, when treated as continuous variables, were significantly associated with cognitive outcomes. Log2-PC was positively correlated with cognitive performance across all models (Model 1: OR = 1.402; Model 3: OR = 1.132, both *p* < 0.0001), suggesting a protective effect of higher platelet counts. Similarly, Log2-LC showed a positive association (Model 3: OR = 1.293, *p* < 0.0001), indicating a potential beneficial impact of lymphocytes. In contrast, higher levels of Log2-NC and Log2-NLR were consistently linked to lower odds of normal cognitive performance (Model 3: OR for NC = 0.644; OR for NLR = 0.781; both *p* < 0.0001), suggesting that neutrophil-driven inflammation may have a detrimental effect. Elevated levels of SII and SIRI were also significantly associated with poorer cognitive performance (Model 3: OR for SII = 0.858; OR for SIRI = 0.891; both *p* < 0.0001). Quartile analyses confirmed these observations, with participants in the highest quartiles of SII and SIRI exhibiting significantly higher odds of cognitive impairment compared to those in the lowest quartiles.

**Table 2:** Association of cognitive performance status with SII and inflammatory indicators.

Exposure	Model 1 OR (95% CI)	*p*-value	Model 2 OR (95% CI)	*p*-value	Model 3 OR (95% CI)	*p*-value
Log2-PC	1.402 (1.398, 1.406)	0.0001	1.105 (1.101, 1.108)	0.0001	1.132 (1.128, 1.135)	0.0001
PC categorical						
Q1	Reference		Reference		Reference	
Q2	1.614 (1.609, 1.619)	0.0001	1.392 (1.387, 1.397)	0.0001	1.331 (1.326, 1.336)	0.0001
Q3	1.666 (1.661, 1.672)	0.0001	1.146 (1.141, 1.150)	0.0001	1.120 (1.116, 1.125)	0.0001
Q4	1.282 (1.278, 1.286)	0.0001	1.009 (1.005, 1.012)	< 0.001	1.062 (1.058, 1.066)	< 0.001
Log2-MC	0.998 (0.996, 1.000)	0.0726	1.136 (1.133, 1.139)	0.0001	1.293 (1.290, 1.297)	0.0001
MC categorical						
Q1	Reference		Reference		Reference	
Q2	1.043 (1.039, 1.046)	< 0.001	1.265 (1.260, 1.269)	0.0001	1.307 (1.302, 1.312)	0.0001
Q3	1.076 (1.072, 1.080)	0.0001	1.404 (1.399, 1.410)	0.0001	1.596 (1.590, 1.603)	0.0001
Q4	0.983 (0.980, 0.986)	< 0.001	1.233 (1.229, 1.238)	0.0001	1.420 (1.415, 1.426)	0.0001
Log2-LC	1.279 (1.276, 1.282)	0.0001	1.263 (1.260, 1.266)	0.0001	1.364 (1.360, 1.368)	0.0001
LC categorical						
Q1	Reference		Reference		Reference	
Q2	1.430 (1.425, 1.435)	0.0001	1.124 (1.120, 1.129)	0.0001	1.085 (1.081, 1.090)	0.0001
Q3	1.623 (1.618, 1.628)	0.0001	1.348 (1.344, 1.353)	0.0001	1.228 (1.224, 1.233)	0.0001
Q4	1.250 (1.246, 1.254)	0.0001	1.260 (1.256, 1.265)	0.0001	1.249 (1.244, 1.254)	0.0001
Log2-NC	0.623 (0.622, 0.624)	0.0001	0.684 (0.682, 0.686)	0.0001	0.644 (0.641, 0.647)	0.0001
NC categorical						
Q1	Reference		Reference		Reference	
Q2	1.433 (1.428, 1.439)	0.0001	1.489 (1.483, 1.495)	0.0001	1.370 (1.364, 1.376)	0.0001
Q3	0.802 (0.800, 0.805)	0.0001	0.928 (0.924, 0.931)	0.0001	0.849 (0.846, 0.853)	0.0001
Q4	0.614 (0.612, 0.616)	0.0001	0.710 (0.708, 0.713)	0.0001	0.652 (0.649, 0.656)	0.0001
Log2-NLR	0.674 (0.673, 0.675)	0.0001	0.717 (0.715, 0.718)	0.0001	0.781 (0.780, 0.783)	0.0001
NLR categorical						
Q1	Reference		Reference		Reference	
Q2	0.797 (0.794, 0.800)	0.0001	0.752 (0.749, 0.755)	0.0001	0.689 (0.687, 0.692)	0.0001
Q3	0.661 (0.659, 0.664)	0.0001	0.592 (0.590, 0.594)	0.0001	0.611 (0.608, 0.613)	0.0001
Q4	0.414 (0.413, 0.416)	0.0001	0.481 (0.479, 0.483)	0.0001	0.535 (0.533, 0.537)	0.0001
Log2-PLR	0.963 (0.961, 0.965)	< 0.001	0.869 (0.867, 0.871)	0.0001	0.873 (0.871, 0.875)	0.0001
PLR categorical						
Q1	Reference		Reference		Reference	
Q2	1.004 (1.001, 1.007)	0.0209	0.868 (0.865, 0.871)	0.0001	0.877 (0.873, 0.880)	0.0001
Q3	1.077 (1.073, 1.080)	0.0001	0.761 (0.759, 0.764)	0.0001	0.762 (0.759, 0.765)	0.0001
Q4	0.926 (0.923, 0.929)	0.0001	0.804 (0.802, 0.807)	0.0001	0.888 (0.885, 0.891)	0.0001
Log2-MLR	0.837 (0.835, 0.838)	0.0001	0.914 (0.912, 0.916)	0.0001	0.980 (0.978, 0.982)	< 0.001
MLR categorical						
Q1	Reference		Reference		Reference	
Q2	1.027 (1.024, 1.031)	< 0.001	1.118 (1.113, 1.122)	0.0001	1.133 (1.129, 1.138)	0.0001
Q3	0.873 (0.870, 0.876)	0.0001	0.935 (0.932, 0.939)	< 0.001	1.013 (1.009, 1.017)	< 0.001
Q4	0.714 (0.712, 0.717)	0.0001	0.776 (0.773, 0.779)	0.0001	0.860 (0.856, 0.863)	0.0001
Log2-PPN	0.855 (0.853, 0.856)	0.0001	0.851 (0.850, 0.853)	0.0001	0.887 (0.885, 0.888)	0.0001
PPN categorical						
Q1	Reference		Reference		Reference	
Q2	1.677 (1.671, 1.684)	0.0001	1.860 (1.853, 1.868)	0.0001	1.790 (1782, 1.797)	0.0001
Q3	1.119 (1.116, 1.123)	0.0001	1.144 (1.140, 1.148)	0.0001	1.052 (1.048, 1.056)	< 0.001
Q4	0.762 (0.760, 0.765)	0.0001	0.733 (0.731, 0.736)	0.0001	0.724 (0.720, 0.727)	0.0001
Log2-SII	0.796 (0.795, 0.797)	0.0001	0.804 (0.803, 0.805)	0.0001	0.858 (0.856, 0.859)	0.0001
SII categorical						
Q1	Reference		Reference		Reference	
Q2	1.187 (1.183, 1.191)	0.0001	0.890 (0.887, 0.894)	0.0001	0.923 (0.919, 0.926)	0.0001
Q3	1.073 (1.069, 1.077)	0.0001	1.025 (1.021, 1.029)	< 0.001	1.036 (1.032, 1.040)	< 0.001
Q4	0.623 (0.621, 0.625)	0.0001	0.557 (0.575, 0.579)	0.0001	0.675 (0.672, 0.678)	0.0001
Log2-SIRI	0.773 (0.772, 0.774)	0.0001	0.831 (0.829, 0.832)	0.0001	0.891 (0.889, 0.892)	0.0001
SIRI categorical						
Q1	Reference		Reference		Reference	
Q2	1.369 (1.364, 1.374)	0.0001	1.428 (1.422, 1.433)	0.0001	1.326 (1.320, 1.331)	0.0001
Q3	1.001 (0.997, 1.004)	0.5921	1.282 (1.277, 1.287)	0.0001	1.301 (1.296, 1.307)	0.0001
Q4	0.631 (0.629, 0.633)	0.0001	0.787 (0.784, 0.790)	0.0001	0.852 (0.849, 0.856)	0.0001

Model 1: No adjustments; Model 2: Adjusted for sex, age, race, marital status, education level, and PIR; Model 3: Adjusted for sex, age, race, marital status, education level, PIR, BMI, DBP, SBP, TCH, RBC, WBC, smoking status, alcohol consumption, diabetes, stroke, depression, general health status, and sleep disorder.

Quartile ranges for inflammatory markers: PC: Q1 (18–184), Q2 (185–219), Q3 (220–255), Q4 (256–681); MC: Q1 (0.10–0.40), Q2 (0.50–0.50), Q3 (0.60–0.60), Q4 (0.70–10.20); LC: Q1 (0.30–1.40), Q2 (1.50–1.70), Q3 (1.80–2.20), Q4 (2.30–49.00); NC: Q1 (0.10–3.10), Q2 (3.20–3.90), Q3 (4.00–4.90), Q4 (5.00–15.00); NLR: Q1 (0.009–1.625), Q2 (1.632–2.188), Q3 (2.190–2.944), Q4 (2.947–30.667); PLR: Q1 (3.82–97.08), Q2 (97.22–121.00), Q3 (121.18–153.53), Q4 (153.57–920.00); MLR: Q1 (0.027–0.233), Q2 (0.233–0.296), Q3 (0.300–0.385), Q4 (0.389–2.263); PPN: Q1 (22.9–623.70), Q2 (624.00–844.00), Q3 (844.20–1176.50), Q4 (1180.00–5380.50); SII: Q1 (1.53–342.86), Q2 (343.33–464.06), Q3 (465.38–680.63), Q4 (680.65–8464.00); SIRI: Q1 (0.056–0.800), Q2 (0.805–1.162), Q3 (1.165–1.768), Q4 (1.770–24.600).

### 3.3 Subgroup analyses of the association between SII, SIRI, and cognitive performance

Subgroup analyses indicated a particularly strong association between SIRI and cognitive performance in males (OR = 0.83, *p* = 0.003), individuals aged 60–79, and those with lower PIR (OR = 0.63, *p* = 0.009), suggesting that biological sex, age, and socioeconomic status may influence the impact of systemic inflammation. While SIRI exhibited consistent associations across subgroups, SII also exhibited significant effects in certain groups, such as males and individuals aged 70–79, with evidence of an age-related interaction (*P*-interaction = 0.026). These results highlight the role of social and biological factors in modulating inflammation-driven cognitive decline (Table 3).

**Table 3:** Subgroup analysis for the association between SII, SIRI, and cognitive performance.

Characteristic	Count	SIRI			SII		
		OR (95%CI)	*p-*value	*P* for interaction	OR (95%CI)	*p*-value	*P* for interaction
Sex				0.547			0.203
Female	1,089	0.80 (0.59 ~ 1.09)	0.160		1 (1 ~ 1)	0.486	
Male	1,105	0.83 (0.73 ~ 0.94)	0.003		1 (1 ~ 1)	0.002	
Age				0.260			0.026
60–69	1,193	0.70 (0.53 ~ 0.92)	0.011		1 (1 ~ 1)	0.103	
70–79	649	0.78 (0.63 ~ 0.97)	0.026		1 (1 ~ 1)	0.002	
≥ 80	352	0.94 (0.78 ~ 1.13)	0.500		1 (1 ~ 1)	0.695	
Race				0.138			0.051
Mexican American	172	0.33 (0.12 ~ 0.87)	0.025		1 (1 ~ 1)	0.027	
Non-Hispanic Black	221	0.75 (0.42 ~ 1.32)	0.317		1 (1 ~ 1)	0.714	
Non-Hispanic White	1,106	0.87 (0.77 ~ 0.99)	0.028		1 (1 ~ 1)	0.025	
Other Hispanic	501	0.76 (0.50 ~ 1.15)	0.196		1 (1 ~ 1)	0.761	
Other races, including multiracial	194	0.78 (0.29 ~ 2.08)	0.617		1 (1 ~ 1)	0.216	
Marital status				0.689			0.421
Married/living with partner	1,295	0.85 (0.74 ~ 0.99)	0.036		1 (1 ~ 1)	0.022	
Widowed/Divorced/Separated	780	0.72 (0.55 ~ 0.94)	0.015		1 (1 ~ 1)	0.094	
Never married	119	1.00 (0.28 ~ 3.57)	0.998		1 (0.99 ~ 1)	0.526	
Education level				0.962			0.639
Below high school	518	0.75 (0.58 ~ 0.95)	0.020		1 (1 ~ 1)	0.204	
High School graduate	518	0.87 (0.65 ~ 1.16)	0.336		1 (1 ~ 1)	0.135	
Above high school	1,158	0.86 (0.74 ~ 0.99)	0.034		1 (1 ~ 1)	0.089	
PIR				0.346			0.539
Low (< 2.23)	542	0.63 (0.45 ~ 0.89)	0.009		1 (1 ~ 1)	0.031	
Middle (2.24–4.28)	1,103	0.83 (0.71 ~ 0.97)	0.022		1 (1 ~ 1)	0.202	
High (> 4.29)	549	0.96 (0.73 ~ 1.28)	0.805		1 (1 ~ 1)	0.602	
BMI				0.354			0.398
Underweight (< 18.5)	30	0.45 (0.22 ~ 0.90)	0.025		1.02(0 ~ 9.45)	1	
Normal (18.5–25)	550	0.89 (0.75 ~ 1.05)	0.167		1 (1 ~ 1)	0.658	
Overweight (25–30)	788	0.83 (0.69 ~ 1.00)	0.051		1 (1 ~ 1)	0.024	
Obese (≥ 30)	826	0.74 (0.57 ~ 0.95)	0.020		1 (1 ~ 1)	0.008	
Smoke				0.361			0.287
Yes	1,122	0.81 (0.71 ~ 0.94)	0.004		1 (1 ~ 1)	0.001	
No	1,072	0.80 (0.65 ~ 0.98)	0.032		1 (1 ~ 1)	0.388	
Alcohol				0.145			0.547
Yes	1,528	0.84 (0.74 ~ 0.95)	0.007		1 (1 ~ 1)	0.056	
No	666	0.75 (0.59 ~ 0.96)	0.022		1 (1 ~ 1)	0.103	
Stroke				0.388			0.206
Yes	146	0.69 (0.43 ~ 1.11)	0.123		1 (1 ~ 1)	0.098	
No	2048	0.87 (0.76 ~ 1.00)	0.043		1 (1 ~ 1)	0.136	
Diabetes				0.068			0.548
Yes	721	0.88 (0.72 ~ 1.07)	0.187		1 (1 ~ 1)	0.162	
No	1,473	0.78 (0.65 ~ 0.94)	0.009		1 (1 ~ 1)	0.039	
Depression				0.536			0.296
Yes	171	0.75 (0.42 ~ 1.34)	0.337		1 (1 ~ 1)	0.088	
No	2023	0.84 (0.75 ~ 0.95)	0.004		1 (1 ~ 1)	0.032	
Sleep disorder				0.236			0.016
Yes	266	0.62 (0.38 ~ 1)	0.051		1 (1 ~ 1)	0.007	
No	1928	0.87 (0.76 ~ 0.99)	0.037		1 (1 ~ 1)	0.200	

### 3.4 SII, SIRI, and cognitive performance: restricted cubic spline plots analysis

**Figure 2:**
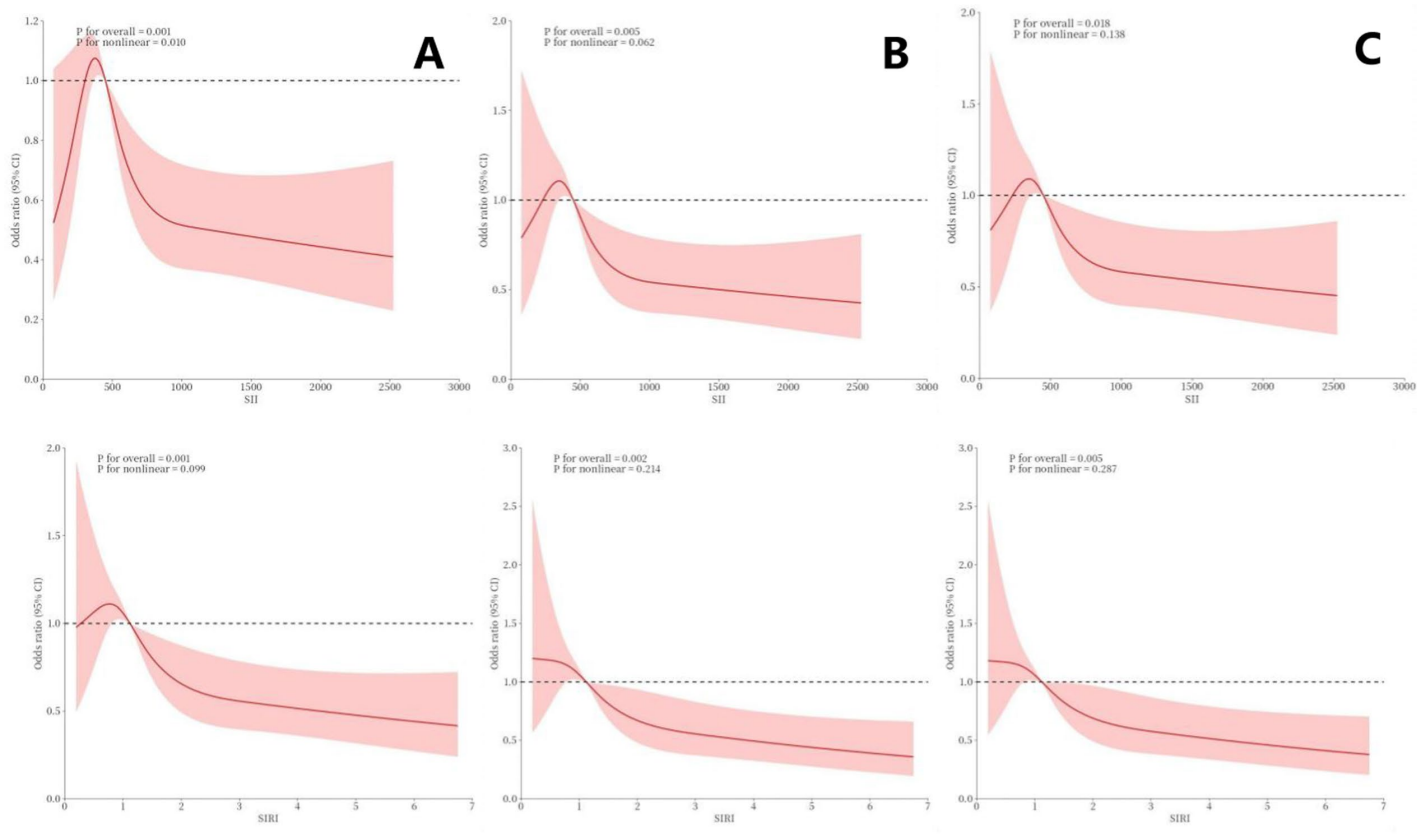
Non-linear relationship between SII, SIRI, and cognitive performance, as assessed using RCS. Panel **(A)** represents Model 0, with no adjustments for confounding factors. Panel **(B)** represents Model 1, with adjustments for age, gender, PIR, BMI, race, education level, and marital status. Panel **(C)** represents Model 2, with adjustments for age, gender, PIR, BMI, race, education level, marital status, diabetes, stroke, sleep disorder, smoking, alcohol use, and depression.

The RCS plots in Figure 2 demonstrate significant non-linear relationships between SII, SIRI, and cognitive performance. In the unadjusted Model 0, both SII and SIRI exhibited notable non-linear associations with cognitive performance (SII: *P* for overall < 0.001, *P* for nonlinearity = 0.010; SIRI: *P* for overall < 0.001, *P* for nonlinearity = 0.009), indicating a complex, potentially threshold-dependent relationship between systemic inflammation and cognitive health. In Model 1, adjusted for demographic and socioeconomic factors, these associations remained significant (SII: *p* = 0.005; SIRI: *p* = 0.002), although the non-linear patterns were less pronounced, suggesting that some of the variation could be explained by these baseline confounders. In the fully adjusted Model 2, which controlled for health-related factors such as diabetes, stroke, sleep disorders, smoking, and alcohol use, the association between SIRI and cognitive performance remained statistically significant (*P*-overall = 0.005), though the non-linearity was less marked. The persistence of this relationship, particularly for SIRI, after comprehensive adjustments, underscores its potential as a robust biomarker for cognitive risk, emphasizing the role of systemic inflammation in cognitive decline beyond traditional demographic and clinical factors.

## 4 Discussion

This study provides novel evidence on the relationship between two composite inflammatory indices—SII and SIRI—and cognitive function in a nationally representative population. Both markers were positively associated with cognitive performance, with SIRI demonstrating a stronger and more consistent correlation. These findings suggest that SIRI may be a more sensitive indicator of cognitive impairment risk, highlighting the role of systemic inflammation in cognitive decline. This association remained robust across various demographic subgroups, as confirmed by subgroup and interaction analyses.

SIRI, SII, and other composite inflammatory markers offer a more refined representation of peripheral inflammation and have been closely linked to central nervous system inflammation and cognitive decline (29, 30). The connection between SII, SIRI, and cognitive function may be explained by mechanisms involving chronic systemic inflammation. Such inflammation has been shown to compromise the blood–brain barrier, triggering neuroinflammation that leads to synaptic dysfunction and neuronal loss, ultimately resulting in cognitive impairment (31, 32). SIRI, which integrates neutrophil and lymphocyte counts, may more accurately reflect this chronic inflammatory response. Elevated neutrophil levels are associated with increased oxidative stress, which may further exacerbate neurodegeneration and cognitive dysfunction (33). In contrast, SII may be more indicative of an acute inflammatory state.

Our findings are consistent with previous studies reporting associations between systemic inflammation and cognitive impairment (34, 35), further supporting the role of inflammation in cognitive decline. The study also underscores the differential impact of SII and SIRI across demographic and socioeconomic subgroups. Notably, SIRI exhibited stronger associations in specific populations, such as males and individuals with lower income levels, suggesting that both biological and social determinants may modulate the effects of inflammation on cognitive function. These results align with the work of David Furman and colleagues, who identified chronic systemic inflammation—driven by lifestyle-related factors—as a key contributor to various diseases, including autoimmune and neurodegenerative disorders (36).

Recent clinical studies highlight the significant role of immuno-bone regulation in neuroinflammation and cognitive decline, in addition to systemic inflammation. This mechanism operates through various levels, including bone-derived factors such as Sclerostin and osteocalcin (OCN), the Wnt signaling pathway, and neuroinflammatory axes like cGAS/STING. For example, in patients with osteoporosis, osteocyte-secreted Sclerostin can cross the blood–brain barrier, inhibit neuronal Wnt/*β*-catenin signaling, and promote β-amyloid (Aβ) accumulation, thus accelerating cognitive decline (37). In response to these pathological mechanisms, novel bioengineering and nanotechnology-based interventions have been developed to modulate the bone microenvironment and regulate bone metabolism for osteoporosis treatment. Bioinspired nanovesicles (BNVs) have been utilized to reprogram the secretory phenotype of bone endothelial cells (38), while extracellular vesicle-based delivery systems derived from mesenchymal stem cells (MSCs) induced from human induced pluripotent stem cells (iPSCs) have been designed for siRNA transport in therapeutic applications (39). Additionally, a engineered cell-membrane-coated nanogels PNG @mR&C, constructed using bone mesenchymal stem cell (BMSC) membranes overexpressing RANK and CXCR4, enables the targeted clearance of nuclear factor-𝜿B ligand (RANKL) within the bone microenvironment and controlled release of PTH 1–34, effectively inhibiting bone resorption and restoring metabolic homeostasis (40).

OCN, a crucial osteoblast-secreted protein, has been shown to alleviate cognitive impairment by reducing amyloid burden and enhancing glycolysis in glial cells (41). Moreover, microglial activation in the central nervous system driven by the cGAS-STING pathway has been identified as a key factor in aging-related chronic inflammation and functional decline (42). Systemic inflammatory markers SIRI and SII may indirectly reflect a broader immunoregulatory mechanism linking bone metabolism and central nervous system function.

Sleep quality plays a pivotal role in cognitive function. Although no statistically significant association between sleep disorders and cognitive performance was identified in this study, residual confounding may account for this lack of association. Nonetheless, prior research has indicated that sleep may serve as a key modulator in the relationship between inflammation and cognition. Sleep deprivation activates brain microglia, triggering the release of pro-inflammatory cytokines that initiate neuroinflammation and accelerate cognitive decline (43–45). Chronic sleep deprivation further promotes systemic inflammation, oxidative stress, and cellular damage, exacerbating neural dysfunction and cognitive impairment (46, 47). A recent study involving both human participants and mouse models found that insufficient sleep activates oxidative stress and integrated stress response pathways in GABAergic neurons, potentially contributing to the onset and progression of neurological disorders (48). As GABAergic neurons are integral to sleep regulation, memory consolidation, and stress responses (49), these findings highlight the critical role of sleep quality in preserving cognitive health.

This study has several limitations. Its observational design prevents causal inference, and unmeasured confounders may be present. While SII and SIRI reflect systemic inflammation, they do not encompass all pathways associated with cognitive decline. Lifestyle factors such as diet and stress were not considered. Future longitudinal studies should track inflammatory markers over time and incorporate broader behavioral and biological variables, including those related to immuno-bone regulation. The observed non-linear relationship between SIRI and cognition warrants further mechanistic exploration.

## 5 Conclusion

This study demonstrates that elevated SII and SIRI levels are significantly associated with an increased risk of cognitive impairment, with SIRI exhibiting greater sensitivity and consistency across various models and subgroups. As an inflammation-based biomarker derived from routine blood tests, SIRI shows practical potential for early identification of individuals at high risk for cognitive decline.

## Data Availability

The original contributions presented in the study are included in the article/supplementary material, further inquiries can be directed to the corresponding author.
